# Supplementary material for: Enzymatic Synthesis and Structural Modeling of Bio-Based Oligoesters as an Approach for the Fast Screening of Marine Biodegradation and Ecotoxicity
Source: Int J Mol Sci. 2024 May 16;25(10):5433. doi: 10.3390/ijms25105433 (PMC11121971; doi:10.3390/ijms25105433)
Supplement: Supplementary file 1 [file ijms-25-05433-s001.zip › ijms-2945669-supplementary.pdf]

International Journal of *Molecular Sciences*

MDPI

Supplementary Materials

Enzymatic synthesis and structural modelling of bio-based oligoesters as an approach for fast screening of marine biodegradation and ecotoxicity

Anamaria Todea [1,2*], Ioan Bîtcan [1,2], Marco Giannetto [1], Iulia Ioana Rădoi [1], Raffaele Bruschi [1,3], Monia Renzi [3], Serena Anselmi [4], Francesca Provenza [4], Tecla Bentivoglio [4], Fioretta Asaro [1], Emanuele Carosati [1] and Lucia Gardossi [1]

[1] Department of Chemical and Pharmaceutical Sciences, University of Trieste, Via L. Giorgieri 1, 34127 Trieste TS, Italy
[2] University Politehnica Timisoara, Faculty of Industrial Chemistry and Environmental Engineering, 300001, C. Telbisz 6, 300001 Timisoara, Romania
[3] Department of Life Sciences, University of Trieste, via L. Giorgieri, 10 34127 Trieste TS, Italy
[4] Bioscience Research Center, via Aurelia Vecchia, 32, 58015 Orbetello, Italy.
* Correspondence: atodea@units.it (A.T.)

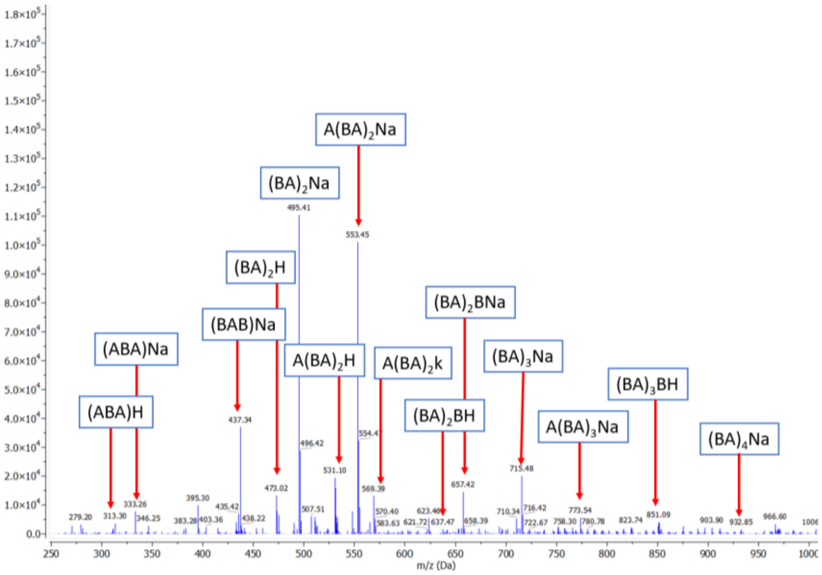

Figure S1. The ESI-MS spectrum of the enzymatically synthesized poly(butylene terephthalate) obtained in *t*-BuOH at 70°C in the presence of Novozyme 435 lipase.

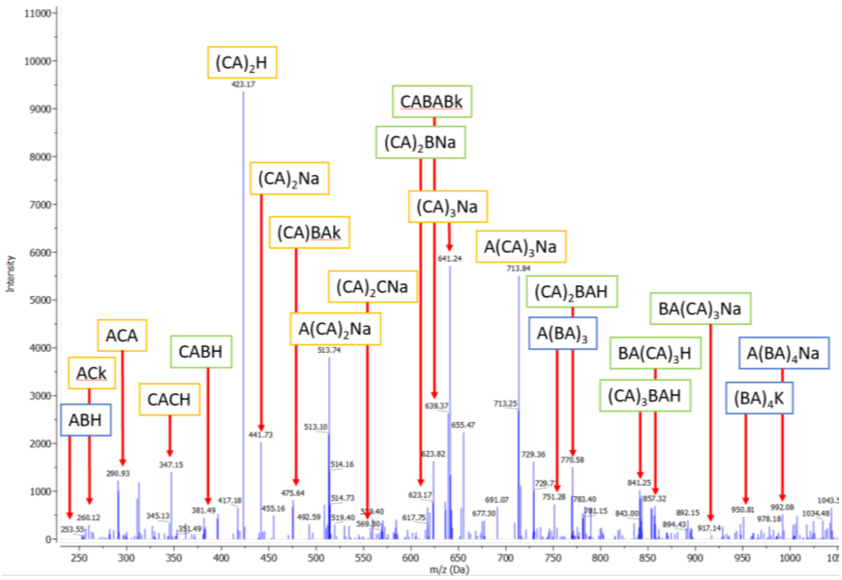

Figure S2. The ESI-MS spectrum of the enzymatically synthesized poly(butylene adipate terephthalate) obtained in *t*-BuOH at 70°C in the presence of Novozyme 435.

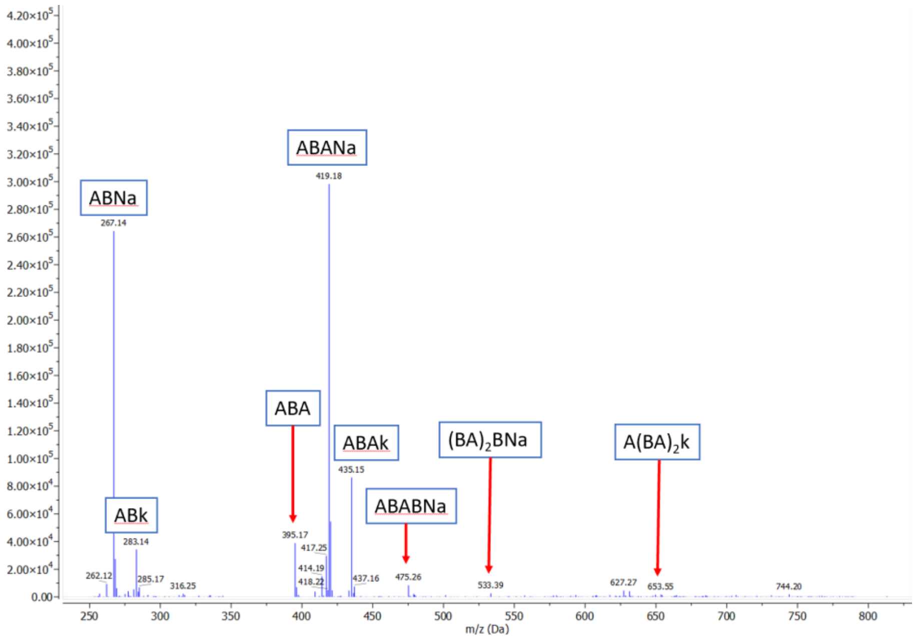

Figure S3. The ESI-MS spectrum of the enzymatically synthesized poly(glycerol furanoate) obtained in *t*-BuOH at 70°C in the presence of covalently immobilized CalB lipase.

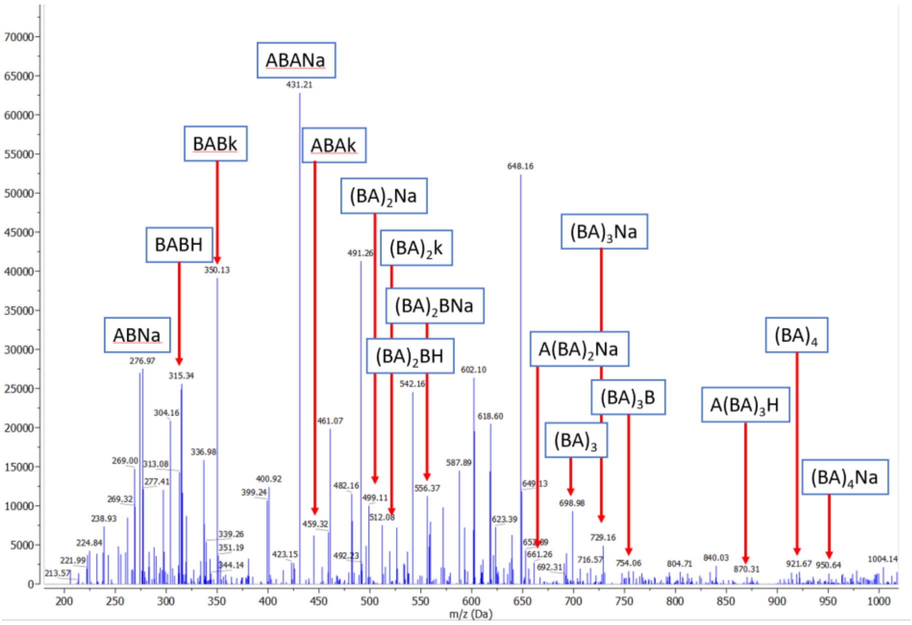

Figure S4. The ESI-MS spectrum of the enzymatically synthesized poly(glycerol terephthalate) obtained in *t*-BuOH at 70°C in the presence of Novozyme 435 lipase.

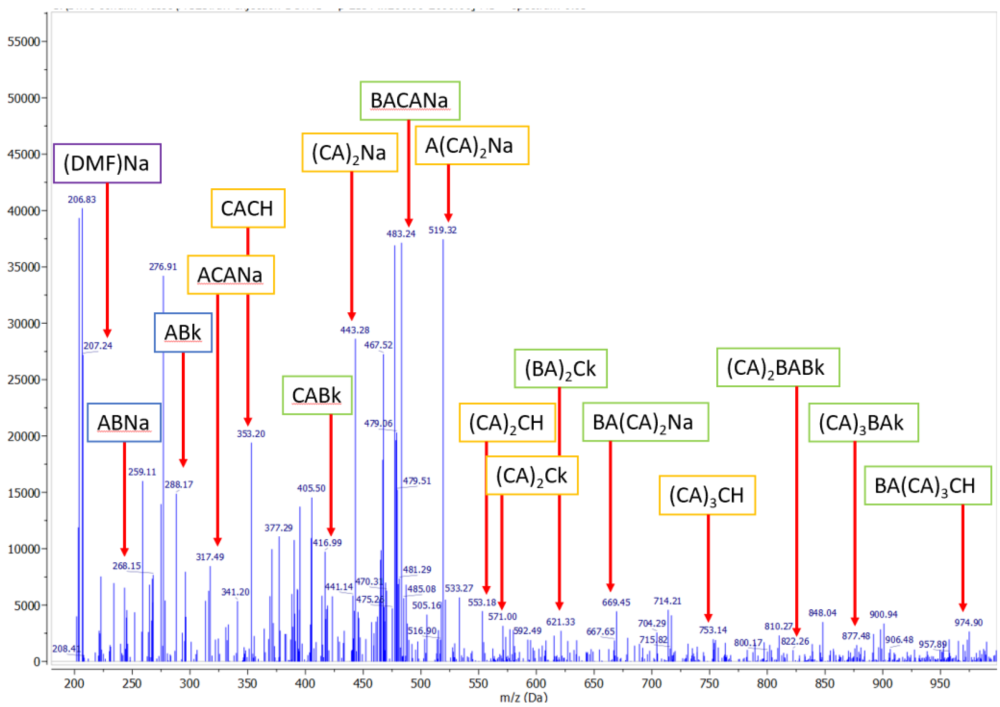

Figure S5. The ESI-MS spectrum of the enzymatically synthesized poly(glycerol adipate furanoate) obtained in *t*-BuOH at 70°C in the presence of covalently immobilized CalB lipase.

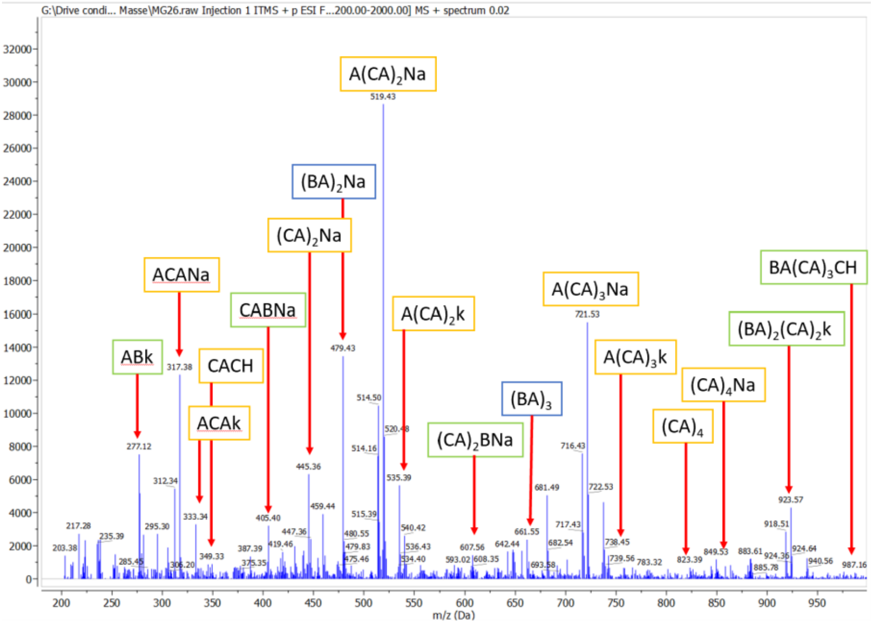

Figure S6. The ESI-MS spectrum of the enzymatically synthesized poly(glycerol adipate terephtalate) obtained in *t*-BuOH at 70°C in the presence of Novozyme 435 lipase.

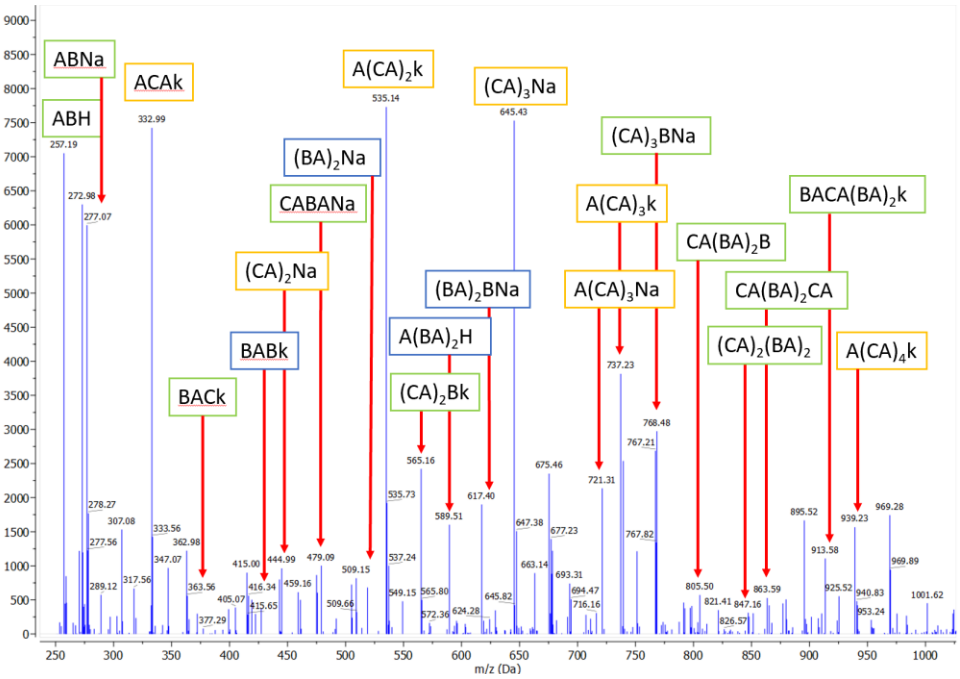

Figure S7. The ESI-MS spectrum of the enzymatically synthesized poly(glycerol erythritol adipate) obtained in *t*-BuOH at 70°C in the presence of Novozyme 435 lipase.

Table S1. The thermal parameters determined by differential scanning calorimetry in comparison with Mn, dispersity, and biodegradability.

Product	T_g [°C]	T_m [°C]	ΔH_{μ} [ϑ/γ]	T_c [°C]	Physical state	M_n [g/mol]	Đ	D_t [%]
Mixtures of ter-oligomers								
BDO TA AA	-21.6	4.5	5.05	96.9	L	1002	1.21	36.48
GLY ERI AA	-28	n.d.	n.d.	amorph	L	844	1.35	30.48
BDO FDCA AA	-43.4	74.4	7.15	amorph	S	844	1.06	29.00

GLY FDCA AA	-31.6	108.9	-22.48	66	L	439	1.25	22.55
GLY TA AA	-48.5	132.3	30.51	123.1	L	474	1.31	19.38

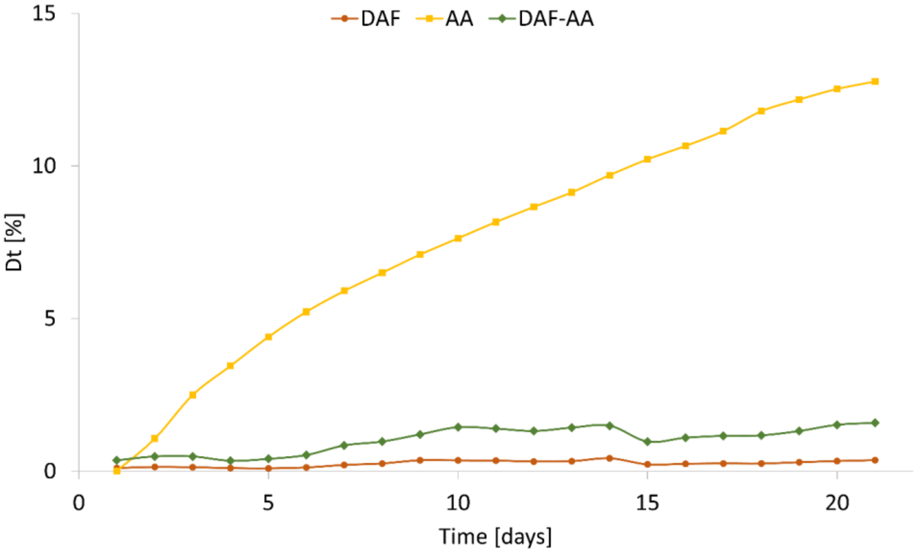

Figure S8. Evaluation of monomer and polyesteramide degradation following a 14-day incubation in a marine environment (the data were normalized by subtracting the values obtained for the control samples).

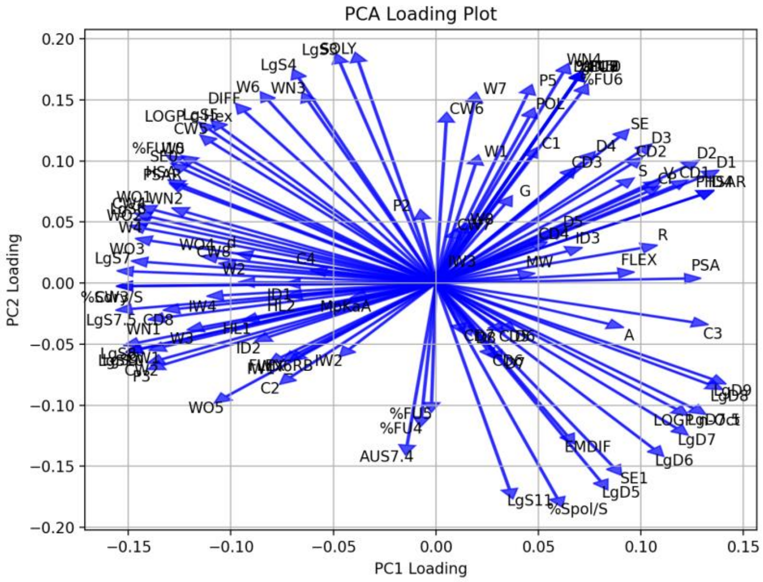

Figure S9. PCA Loading plot obtained by using Volsurf3.

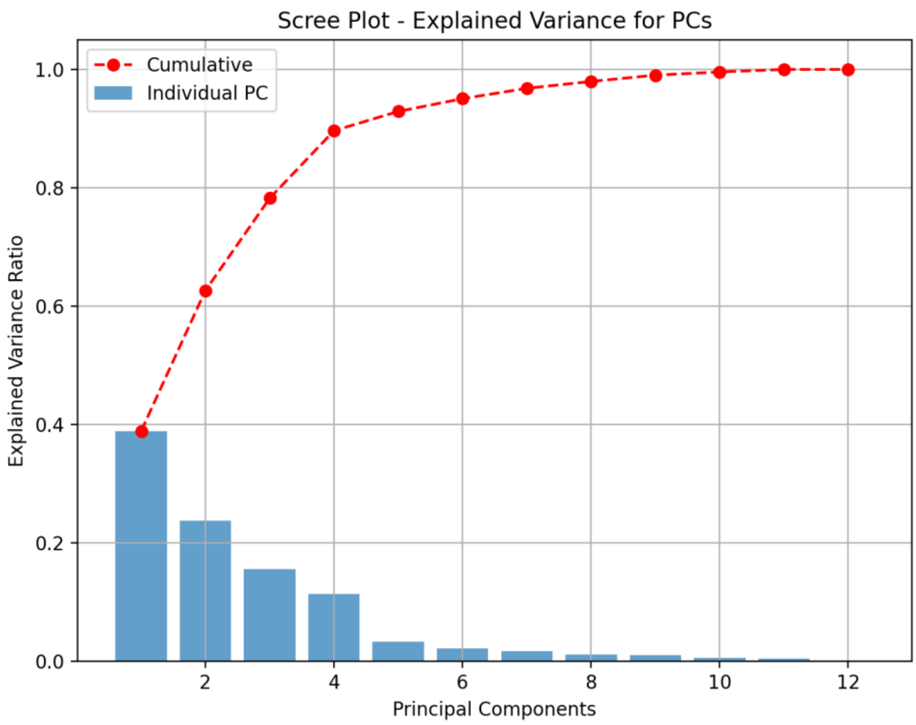

Figure S10. The Scree Plot- Explained variance plot for PCs.

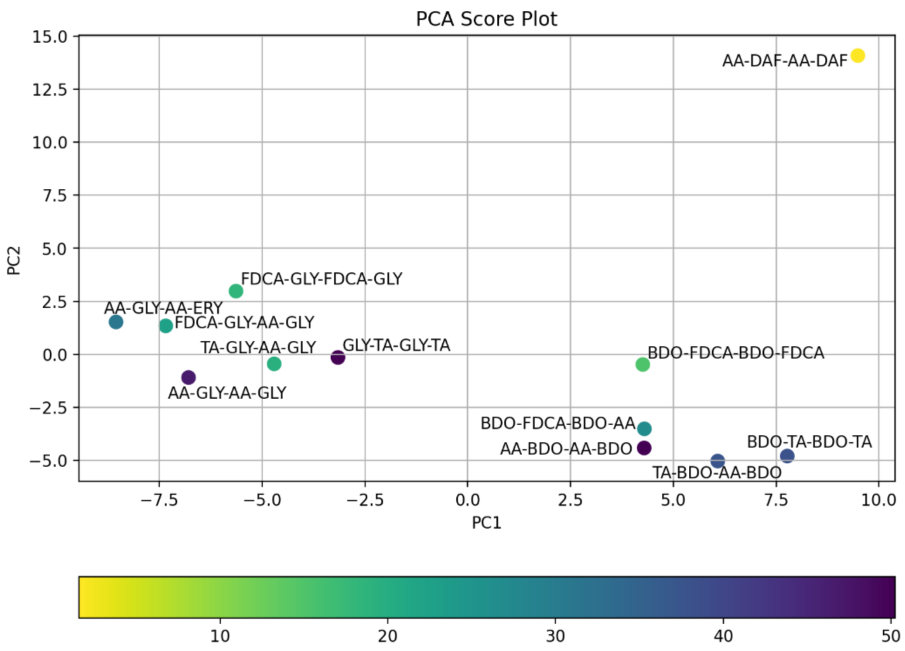

Figure S11. PCA score plots PC1 vs PC2, with objects coloured by biodegradation.

Table S2. PLS: data for fitting and validation of the model.

LV	R2 (fitting)	Q2 (LOO-validation)
1	0.47	Neg
2	0.60	0.02
3	0.83	Neg

4	0.95	0.17
**5**	**0.98**	**0.57**

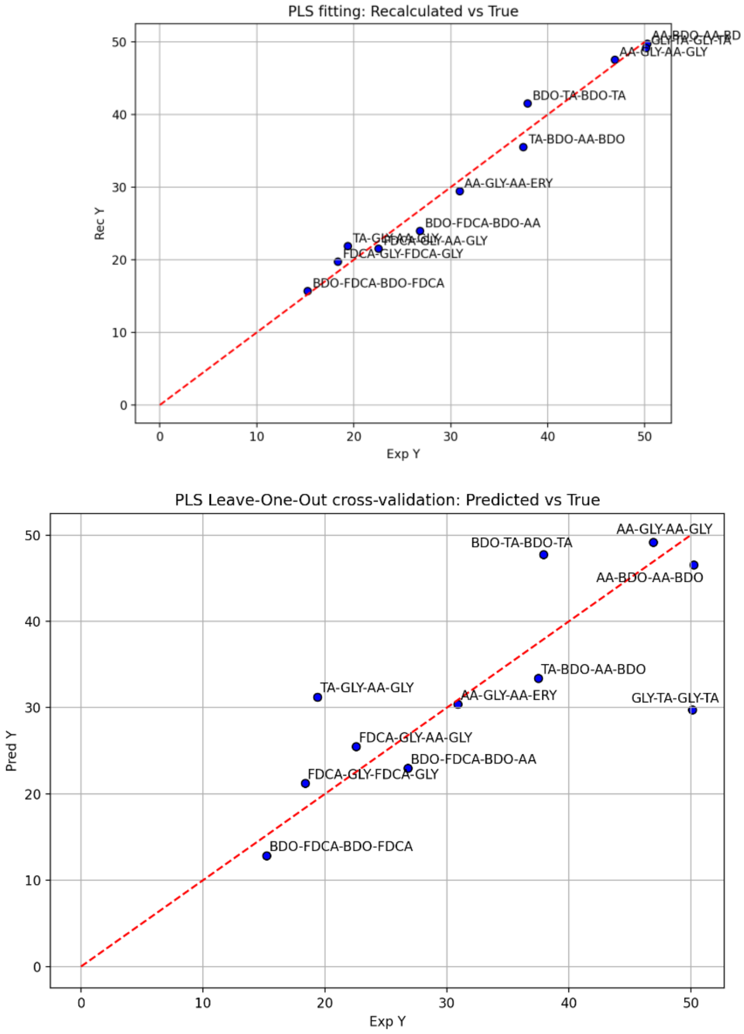

Figure S12. Recalculated and predicted biodegradation values for the PLS model based on 5 latent variables from 18 VolSurf[3] descriptors.

Table S3. A brief description of the Volsurf relevant descriptors for the present study.

Volsurf+ descriptors	Description	Probe used for descriptor calculation
**LogP octanol/water (LOGP n-Oct)**	The logarithm of the partition coefficient between 1-octanol and water is computed via a linear equation derived by fitting GRID-derived atom type to experimental data on n-octanol/water partition coefficients.	other
**Integy moment (IW1-IW4)**	Express the unbalance between the centre of mass of a molecule and the barycenter of its hydrophilic or hydrophobic regions,	OH2
**Capacity Factor (CW1-CW8)**	Represent the ratio of the hydrophilic volume over the total molecular surface and are calculated at eight different energy levels, the same levels used to compute the hydrophilic volumes.	OH2
**Polar and Hydrophobic Surface Areas (PSA, HSA)**	The Polar Surface Area (PSA) is calculated via the sum of polar region contributions, while the Hydrophobic Surface Area (HSA) is calculated via the sum of hydrophobic region contributions	OH2, dry, O, N1

**EMDIF, EMDIS**	Based on GRID potential (dEmin) and atom types, atoms are classified as polar or apolar; thus, differences and geometric distances are calculated.	electronic descriptors
**MpKa**	Most acidic pKa value.	other

Table S4. The ecotoxicity test results obtained in the presence of *Aliivibrio fischeri*.

**Sample**	***Aliivibrio fischeri***			
	**I% 15'**	**SD**	**I% 30'**	**SD**
BDO-FDCA-AA	26.5	2.6	21.7	2.0
BDO-TA-AA	72.0	0.6	68.3	0.6
AA-GLY-ERI	-5.1	0.4	-3.0	0.6
AA-GLY-FDCA	15.7	1.3	15.5	1.8
BDO-FDCA	-2.5	1.6	-2.1	2.2
GLY-FDCA	6.7	0.4	7.7	1.0
GLY-AA	2.6	2.8	5.3	2.9
AA-BDO	10.0	3.2	11.2	2.4
GLY	-1.1	3.0	0.5	2.9
ERI	1.5	2.5	4.0	2.2
FDCA	10.2	2.1	10.8	5.0
BDO	8.4	3.7	9.1	2.9
AA	5.7	4.9	6.8	0.9
TA				

Table S5. The ecotoxicity test results obtained in the presence of *Phaeodactylum tricornutum*.

**Sample**	***Phaeodactylum tricornutum***	
	**I% 72 h**	**SD**
BDO-FDCA-AA	-77.5	2.5
BDO-TA-AA	-14.5	7.1
AA-GLY-ERI	-25.9	5.8
AA-GLY-FDCA	-21.2	4.7
BDO-FDCA	-70.6	3.9
GLY-FDCA	-12.5	0.0
GLY-AA	-33.4	5.8
AA-BDO	-69.2	4.7
GLY	-44.3	4.6
Erythritol	-63.7	3.1
FDCA	-25.2	2.8
BDO	-30.2	6.3
AA	-33.5	3.5
TA	-75.5	2.1

Table S6. The ecotoxicity test results obtained in the presence of *Paracentrotus lividus*.

**Sample**	***Paracentrotus lividus***		
	**Mean**	**SD**	**Adj. Mean**
BDO-FDCA-AA	100.0	0.0	100.0
BDO-TA-AA	100.0	0.0	100.0
AA-GLY-ERI	55.0	1.0	45.3
AA-GLY-FDCA	41.0	1.0	28.3

BDO-FDCA	100.0	0.0	100.0
GLY-FDCA	94.0	1.0	91.5
GLY-AA	100.0	0.0	100.0
AA-BDO	100.0	0.0	100.0
GLY	80.0	3.0	71.6
Erythritol	39.7	1.5	14.2
FDCA	63.0	1.0	47.4
BDO	91.3	0.6	87.7
AA	61.7	1.5	45.5
TA	8.0	1.5	0.0

Table S7. The ecotoxicity test results obtained in the presence of *Daphnia magna.*

Sample	***Daphnia magna***			
	**Mean (24h)**	**SD (24h)**	**Mean (48h)**	**SD (48h)**
BDO-FDCA-AA	0.0	0.0	0.0	0.0
BDO-TA-AA	0.0	0.0	20.0	0.0
AA-GLY-ERI	0.0	0.0	30.0	14.1
AA-GLY-FDCA	0.0	0.0	90.0	14.1
BDO-FDCA	0.0	0.0	20.0	0.0
GLY-FDCA	10.0	14.1	20.0	0.0
GLY-AA	20.0	0.0	20.0	0.0
AA-BDO	10.0	14.1	20.0	0.0
GLY	0.0	0.0	0.0	0.0
ERI	60.0	0.0	100.0	0.0
FDCA	100.0	0.0	100.0	0.0
BDO	10.0	14.1	10.0	14.1
AA	100.0	0.0	100.0	0.0
TA	0.0	0.0	0.0	0.0

Table S8. The ecotoxicity test results obtained in the presence of *Raphidocelis subcapitata.*

Sample	***Raphidocelis subcapitata***	
	**I% 72 h**	**SD**
BDO-FDCA-AA	-11.4	2.4
BDO-TA-AA	18.4	1.4
AA-GLY-ERI	3.1	2.7
AA-GLY-FDCA	1.4	2.4
BDO-FDCA	-25.3	46.8
GLY-FDCA	7.9	3.3
GLY-AA	1.4	6.0
AA-BDO	28.9	3.5
GLY	2.1	5.3
ERI	-2.9	1.2
FDCA	41.9	8.4
BDO	-4.4	4.0
AA	23.4	5.0
TA	-10.3	2.4
